# An Unusual Presentation of Neonatal Sepsis as Hyperleukocytosis With Firm Lymphadenopathy: A Diagnostic Challenge

**DOI:** 10.7759/cureus.30454

**Published:** 2022-10-19

**Authors:** Shikha M Kakkat, Mahaveer S Lakra, Bhavana Lakhar, Apoorv Jain, Rasagnya M Reddy

**Affiliations:** 1 Department of Pediatrics, Jawaharlal Nehru Medical College, Wardha, IND; 2 Department of Neonatology, Jawaharlal Nehru Medical College, Wardha, IND

**Keywords:** reactive lymphadenopathy, toxic granules, leukemoid reaction, newborn, hyperleukocytosis

## Abstract

The neonatal leukemoid reaction is an acute response of the body to stress. Any inflammatory processes in the newborn period may lead to an increase in the white blood cell (WBC) count. Hyperleukocytosis refers to an extremely elevated leukocyte count beyond 100,000/cubic millimeter (cumm). Here, we report a case of a leukemoid reaction in a newborn who presented with fever, swelling over the neck, and failure to thrive. Peripheral smear showed the presence of all precursors of white blood cells, but no blast cells were seen. Fine needle aspiration cytology (FNAC) did not show any abnormal cells or any evidence of leukemia. Hence, the diagnosis of a leukemoid reaction was made. Hyperleukocytosis presenting as palpable lymphadenopathy in a neonate is a rare finding that was seen in this case secondary to septicemia.

## Introduction

Leukocytosis is defined as a rise in the white blood cell (WBC) count in the body as a reaction to an infection or inflammation. Physiological leukocytosis in newborns is characterized by a white blood cell count ranging from 9,000 to 30,000/cubic millimeter (cumm) [1]. A leukemoid reaction is an abnormal proliferation of cells to more than 30,000/cumm due to acute inflammatory response in the newborn period. Hyperleukocytosis is the state where white blood cell counts are more than 100,000/cumm. The overall incidence of a leukemoid reaction is less in the newborn period (around 15%) [2,3]. Holland and Maurer in 1963 reported the first cases of a leukemoid reaction in neonates. The cause of the same includes infection, prematurity, bronchopulmonary dysplasia, asphyxia, chromosomal abnormalities, and steroid use of the mother antenatally [4,5]. The various close differentials are severe septicemia, myeloproliferative disorder, leukocyte adhesion defect, transient abnormal myeloproliferative disorder, leukemia, and abnormal cell proliferation in Down syndrome. In every case of a leukemoid reaction, the cause needs to be investigated.

Lymphadenopathy in a neonate is not a usual finding. An abnormal site, diffuse nature, abnormal size, and consistency in lymphadenopathy should warrant urgent investigation to rule out malignancy [6,7]. Firm lymphadenopathy with acute infection is a rare entity in neonates. Septicemia can have varied presentations in neonates.

Here, we discuss a case where a neonate is presenting with fever, vomiting, failure to thrive, and firm lymphadenopathy. We initially attributed this lymph node to some focus of malignancy as the neonate was having fever, high white blood cell count, and lymphadenopathy but finally came down to the diagnosis of septicemia presenting as a leukemoid reaction with lymphadenopathy.

## Case presentation

A 3.2 kg male neonate, second in birth order, born at 38 weeks of gestation via normal vaginal delivery, had an uneventful delivery. The mother had no significant comorbidities and was not on any regular medications during her pregnancy. Her last complete blood count (CBC) at the time of delivery was suggestive of hemoglobin of 10 g%, total leukocyte count of 24,000 cumm, and platelet count of 3.15 lakhs. The maternal blood group was A positive. Both mother and the neonate had an uneventful postnatal period and were discharged after four days. The neonate allegedly presented multiple times to the medical care unit with a history of vomiting and feeding intolerance. After preliminary tests, he was discharged with parental reassurance. Unhygienic bottle-feeding practices of the parents were discouraged. Such episodes continued initially until the neonate had prolonged episodes of feed intolerance. On the 23rd day of life, he presented at our center with a history of fever, vomiting, loose motion, swelling over the neck, increased work of breathing, and cyanosis for five days.

**Figure 1:**
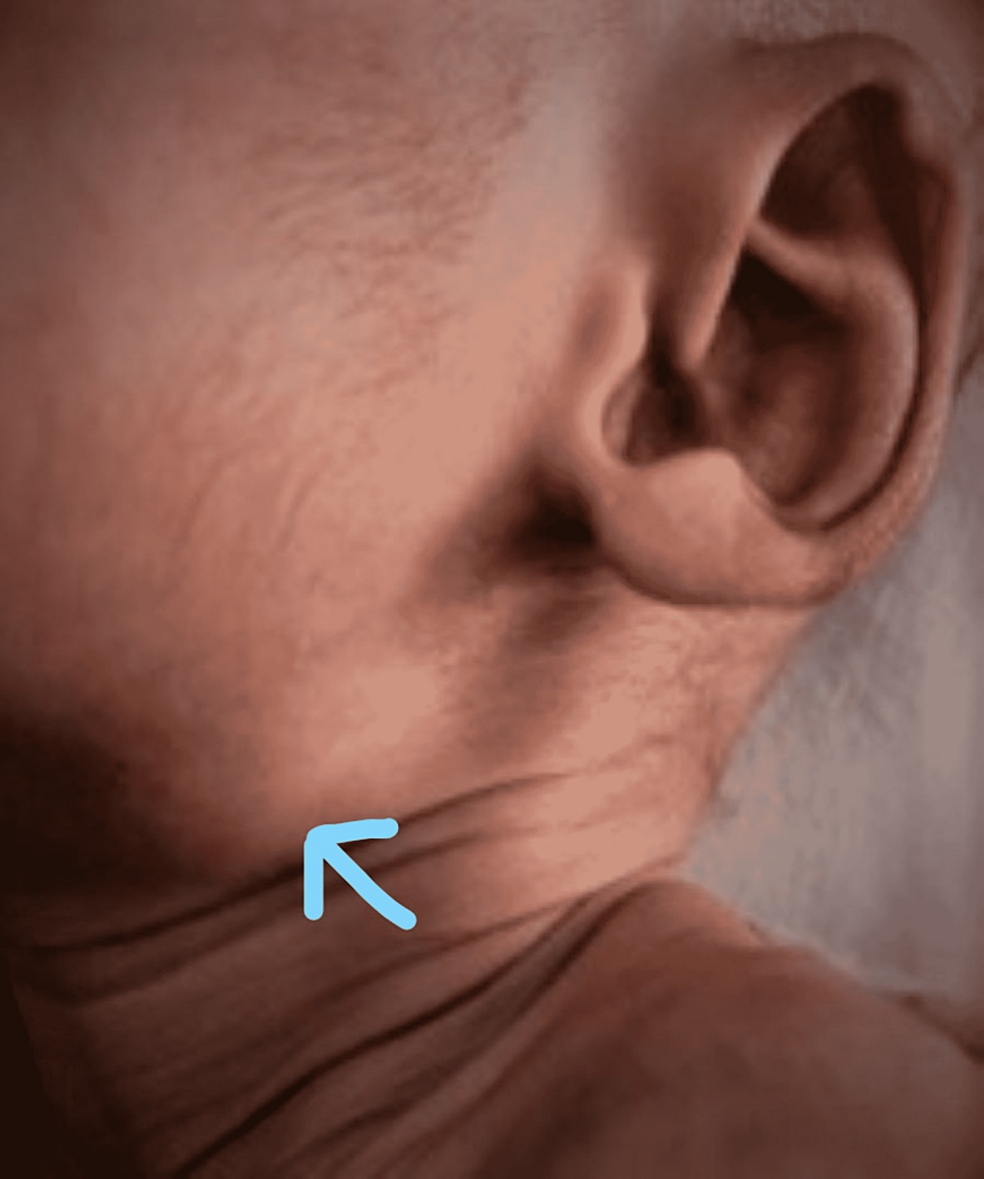
A small left-sided submandibular lymph node (arrow) measuring 2.2 × 1.2 cm that is non-tender, mobile, and firm in consistency.

On examination, the patient was conscious and active with a good tone. His weight on admission was 2.5 kg, temperature was 99.2°F, heart rate was 160 beats/minute, respiratory rate was 54 cycles/minute, 90% SpO_2_ with normal capillary refilling time, and blood pressure was 64/42 mm Hg, which was normal. There was a small left-sided submandibular lymph node present measuring 2.2 × 1.2 cm that is non-tender, mobile, and firm in consistency (Figure 1).

**Figure 2:**
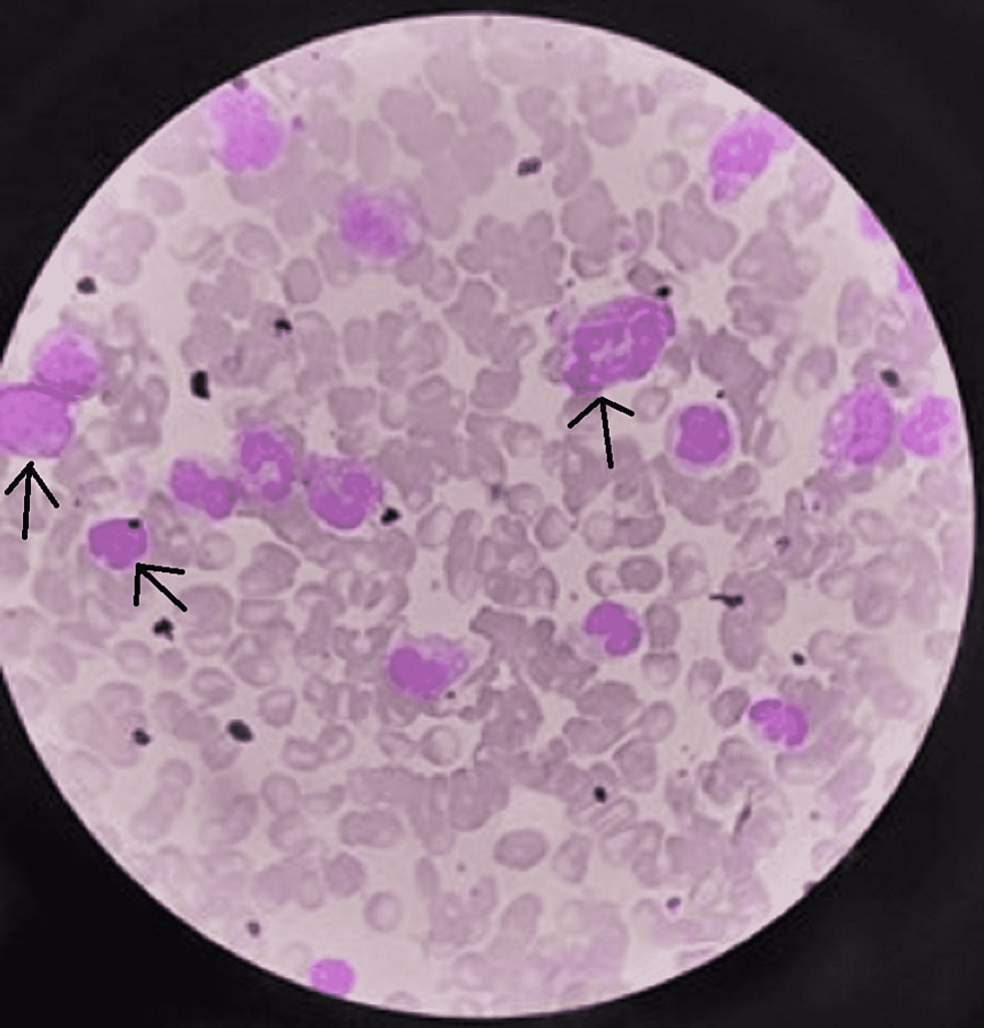
Peripheral smear showing neutrophils shifting to the left up to the promyelocytic stage (arrows). Many neutrophils show toxic granules.

No other lymph nodes were palpable in any other part of the body. Oral examination and throat examination was normal. His cry was normal. The liver was just palpable, and the spleen was also palpable just below the costal margin. Sclerema was present over bilateral lower limbs. Respiratory, cardiovascular, and central nervous system (CNS) examinations were normal. The patient was having dusky skin color with low saturation; therefore, the possibility of septicemia and congenital heart disease was considered. The patient was started on oxygen by nasal prongs, intravenous fluids, antibiotics (cefotaxime and amikacin) to give broad gram-positive and gram-negative bacterial coverage, and other supportive treatment. Blood investigations showed a total leukocyte count (TLC) of 106,000/cumm, 56% neutrophils, 42% lymphocytes, 1% eosinophils, and normal basophils and monocytes. Blood sugar was 94 mg/dL, platelet count was 364,000 per cumm, C-reactive protein (CRP) was 104 mg/dL, blood urea was 58 mg/dL, and creatinine was 0.8 mg. Serum sodium was 158 meq/L, potassium was 5.9 meq/L, and lactate dehydrogenase (LDH) was 616 U/L. Peripheral smear showed increased WBC count, shifting from neutrophils to the left side with toxic granules. The myeloblast was 0%, promyelocyte 17%, myelocyte 0.5%, and metamyelocyte 5%, with no blast cells (Figure 2).

**Figure 3:**
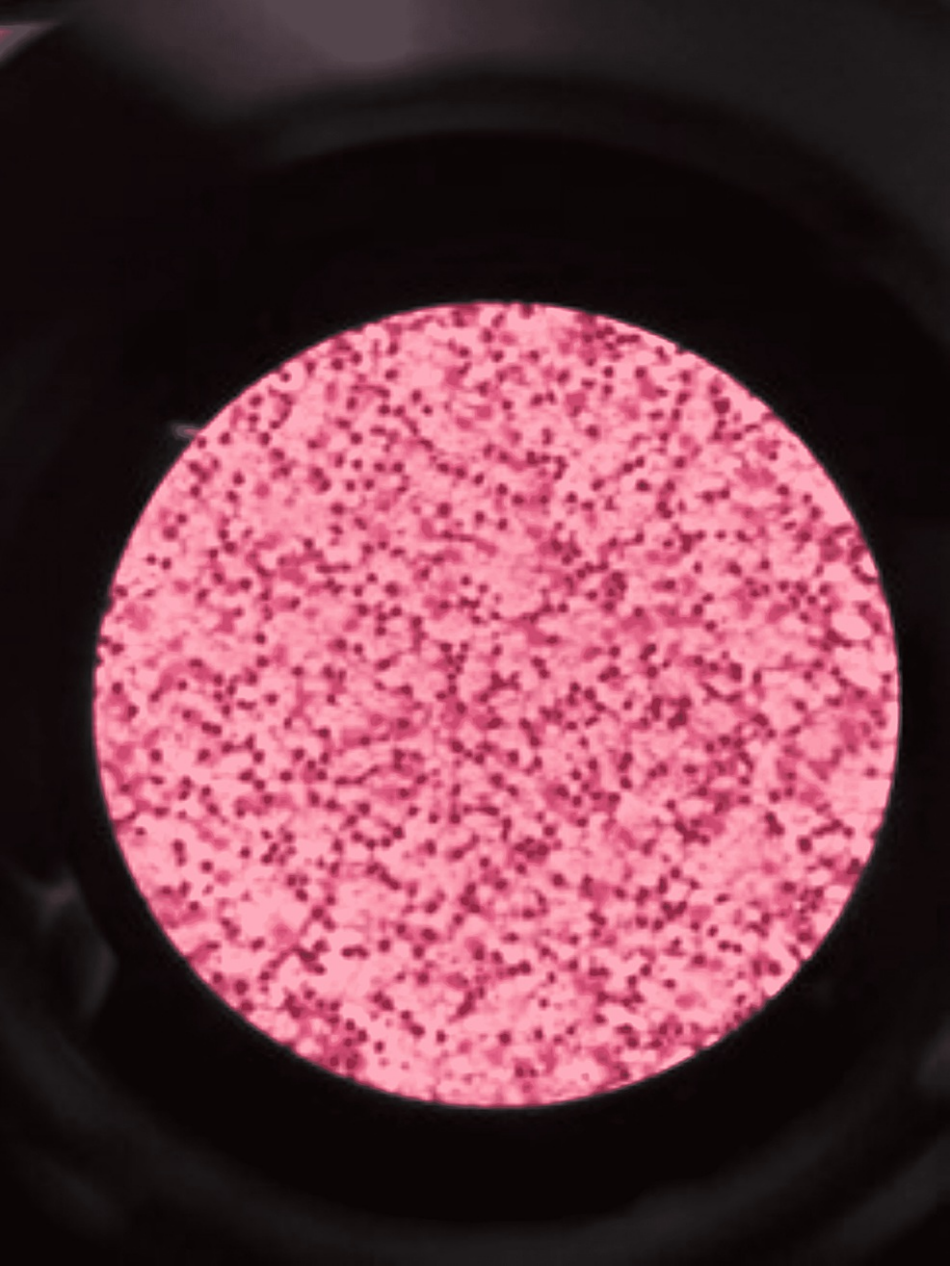
FNAC suggestive of acute inflammatory leukocytic exudation. FNAC: fine needle aspiration cytology

The fundus examination, ultrasonography (USG) of the abdomen and pelvis, and neurosonography were normal to grossly exclude manifestations of the toxoplasmosis, other agents, rubella, cytomegalovirus, and herpes simplex (TORCH) group of infections. A two-dimensional (2D) echo was done to rule out congenital heart defects, which was normal. Suspecting late-onset sepsis, a cerebrospinal fluid study was done, which shows normal WBC, serum protein of 120 mg/dL, and glucose of 75 mg/dL, with LDH of 67 U/L. USG of the lymph node showed evidence of reactive lymphadenopathy, and fine needle aspiration cytology (FNAC) was also suggestive of the same. There were no abnormal cells in the peripheral smear and FNAC (Figure 3).

As malignancy and heart disease were ruled out, the possibility of a leukemoid reaction to septicemia was suspected. A blood culture sent on the day of admission showed growth of *Klebsiella pneumoniae* sensitive to meropenem after 48 hours of culture. Antibiotics were upgraded to meropenem. The repeat blood count was suggestive of a TLC of 41,500/cumm. Serial WBCs were suggestive of decreasing total leukocyte counts. The patient responded well to higher antibiotics. Sclerema reduced and eventually disappeared over 10 days. The patient was weaned off oxygen, and gradual feeding was established. Antibiotics were continued for 21 days. The patient was discharged on the 48th day of life with a weight of 2.45 kg. The patient was followed up after 14 days in a high-risk clinic and was feeding well with adequate weight gain. The patient was then lost to follow-up.

## Discussion

A leukemoid reaction is the abnormal reversible proliferation of the white blood cell where immature neutrophil precursors are stimulated and released into circulation. It could be secondary to infection or any inflammatory process. It is very difficult to differentiate it in its initial stages from premalignant conditions such as transient abnormal myelopoiesis (TAM), myeloproliferative dysplastic syndromes, and leukemia. Cases have been reported in the literature of myeloproliferative disorder and transient abnormal myeloproliferative disorder in neonates. Transient myeloproliferative disorder is now said to be a premalignant condition mostly associated with Down syndrome and may turn into frank leukemia [8]. A case of a leukemoid reaction was reported by Wolfe et al. where a baby developed rashes and the child worsened over time and ultimately landed into leukemia [9]. The clinical presentation, response to antibiotics, absence of central nervous system involvement and hepatosplenomegaly, and absence of blast cells in peripheral smear are the differentiating points of a leukemoid reaction from leukemia. However, there is also a possibility of a leukemoid reaction turning into a malignancy; therefore, any neonate with abnormally elevated leukocyte counts needs to be thoroughly investigated and followed up.

Hyperleukocytosis is an extreme form of a leukemoid reaction with TLC > 100,000/cumm usually occurring as a component of leukemia, TAM, and myeloproliferative disorders. Neonates with hyperleukocytosis should be evaluated for complications such as acute respiratory failure, pulmonary hemorrhage, CNS infarction, hemorrhage, splenic infarction, myocardial ischemia, and renal failure due to renal vessel leukostasis. In our case, hyperleukocytosis was probably a leukemoid reaction secondary to sepsis.

The most common association of a leukemoid reaction is with sepsis. A case was reported by Anguiano Sánchez et al. of a preterm baby who was admitted to the neonatal intensive care unit (NICU) for 17 days in view of prematurity [10]. During his NICU stay, the baby developed septicemia, following which his counts were suggestive of a leukemoid reaction. A case of leukemoid reaction following herpes infection was reported by Underwood et al. in a baby [11]. In our case, also, the cause of hyperleukocytosis was secondary to bacterial sepsis.

Some cases have also been found to be associated with necrotizing enterocolitis. The exact mechanism is not clear but is said to be multifactorial. Infection by bacteria, fungi, and virus stimulates the toll-like receptors, leading to the activation of proteins causing the development of the inflammatory cascade process. The release of cytokine cells and the stimulation and activation of granulocyte colony-stimulating factors and neutrophils are the possible factors concerned with leukemoid reaction.

In a study done by Duran et al., it was found that the overall incidence of neonatal leukemoid reactions is 1.4% [12]. It was found that neonatal leukemoid reaction is associated with a fourfold rise in the incidence of sepsis, a 54-fold rise in bronchopulmonary dysplasia, a sixfold rise in mortality, and a 20-fold rise in intraventricular hemorrhage. The neonatal leukemoid reaction was more commonly associated with premature babies, neonates of mothers with chorioamnionitis, and mothers with early rupture of membranes.

Therefore, it is very important to differentiate between leukemoid reaction and acute leukemia in neonates, which can be differentiated by the presence of hepatosplenomegaly, rashes, abnormal blast cell in peripheral smears, and increased LDH level in leukemia. If a neonate presents with features of septicemia and increased white blood cells, then we should suspect a leukemoid reaction, but leukemoid reaction sometimes eventually turns malignant. Therefore, the neonate must be monitored, and malignancy should be ruled out. In our case, despite hyperleukocytosis and lymphadenopathy, no evidence of malignancy was found, and the patient responded well to antibiotics; hence, it was an abnormal presentation of late-onset septicemia.

## Conclusions

A leukemoid reaction can be a varied presentation of septicemia and inflammatory response in neonates. It is very necessary to keep a close watch on this entity through clinical examination and repeated blood parameters. It must be differentiated from malignancy to decide on appropriate management and prognostication. A differential diagnosis of neonatal sepsis should always be kept in mind when hyperleukocytosis and firm lymphadenopathy coexist with malignancy being ruled out.
